# Advancing Lumbar Disc Herniation Classification from MRI Using an Efficient and Reproducible Classical Machine Learning Framework

**DOI:** 10.3390/diagnostics16203288

**Published:** 2026-10-10

**Authors:** Abdullah Alanazi, Meshari Alazmi, Abdullah Alshammari, Amirah F. Alshammeri

**Affiliations:** 1 College of Computer Science and Engineering, University of Ha’il, Ha’il 81411, Saudi Arabia; s202309667@uoh.edu.sa (A.A.);; 2 Diagnostic Radiology Department, College of Medicine, University of Ha’il, Ha’il 81422, Saudi Arabia

**Keywords:** lumbar disc herniation, magnetic resonance imaging (MRI), machine learning, principal component analysis (PCA), image preprocessing, smoothing, intensity normalization, feature standardization, extra trees, random forest, nested cross-validation

## Abstract

**Background/Objectives:** A common diagnosis for lumbar disc herniation is an image taken with Magnetic Resonance Imaging (MRI). Due to the need for expertise during manual interpretation of MRI scans by specialists, the process is very time consuming. The use of a machine learning (ML)-based pathway to classify lumbar MRI images as either “normal” or “herniated” represents an efficient way to analyse MRI scans seeking similar pathologies. **Methods:** The proposed methodology transforms original lumbar MRIs (from colour to greyscale), resizes them, filters, and normalises them (using bilateral algorithms) before conducting extraction of features using PCA-based decomposition. A total of ten classical ML classifiers were evaluated using five-fold outer and three-fold inner nested cross-validation, with the number of PCA components selected exclusively within the inner folds. **Results:** Extra Trees achieved the highest F1-score of 85.86% (95% CI: 84.16–87.44%) and the highest ROC-AUC of 90.96% (95% CI: 89.27–92.54%), with an accuracy of 84.29%, precision of 82.03%, recall of 90.07%, and specificity of 77.79%. Random Forest achieved the highest accuracy of 84.36% (95% CI: 82.40–86.26%), although its overall performance was comparable to that of Extra Trees. **Conclusions:** The findings support the potential of traditional machine learning classifiers for binary classification within the evaluated image-level dataset, while maintaining a relatively simple design and computational efficiency. No direct empirical comparison with deep learning models was conducted in this study.

## 1. Introduction

**Figure 1:**
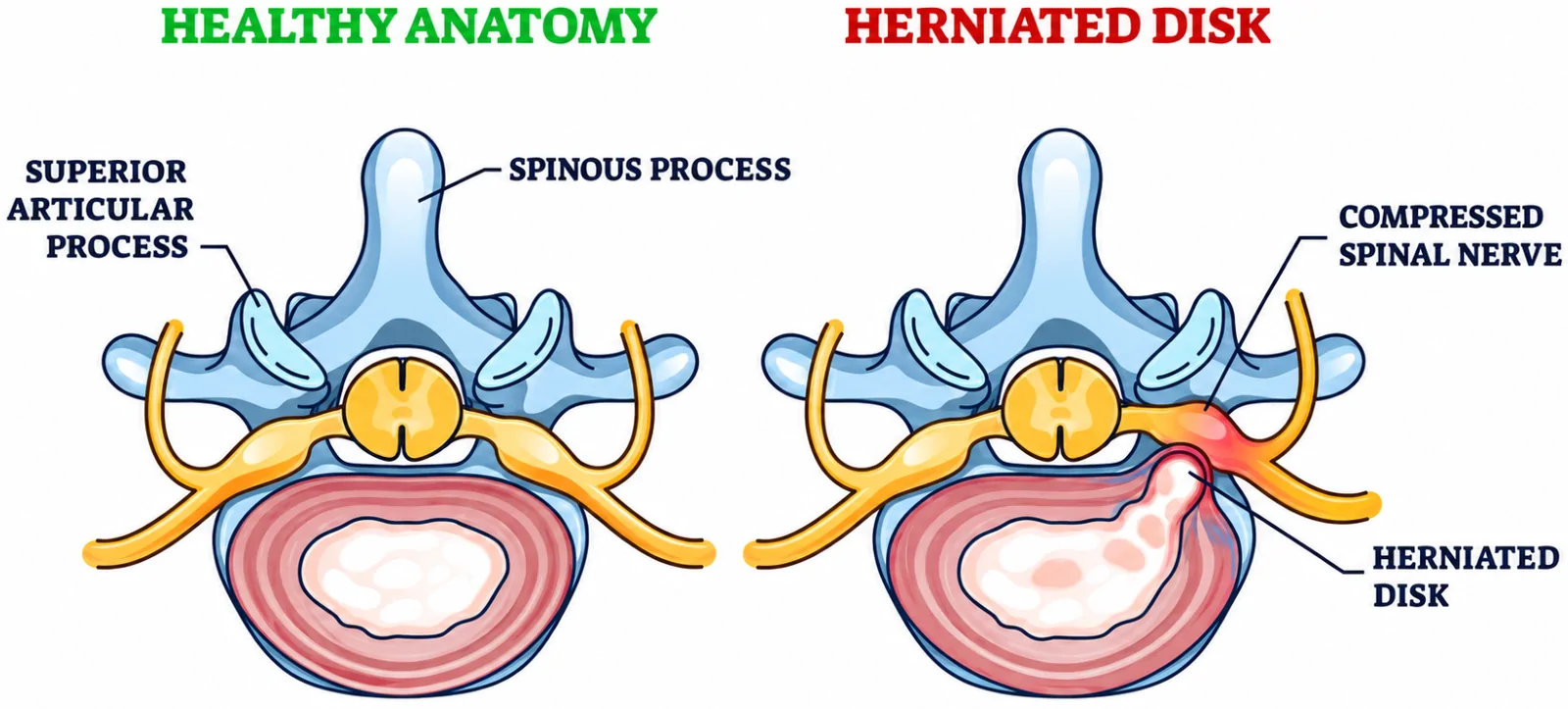
A normal intervertebral disc and a herniated disc with nerve compression in lumbar spine anatomy.

Herniated lumbar discs are a common condition that causes lower back pain on a global scale. Herniation happens when material from the intervertebral disc bulges past its normal limits, which can cause compression on nearby nerves, resulting in pain, numbness, or impaired function. MRI is the gold standard for diagnosing lumbar disc herniation due to the superior contrast of MRI for soft tissues as well as non-invasiveness; however, MRI interpretation can be labour- and expertise-intensive due to the volume of interpretations required. Recent advances in artificial intelligence (AI) now allow for the automated analysis of medical images. CNNs (i.e., deep learning networks) have demonstrated significant improvement in MRIs of the spine. Comparative performance between deep learning and traditional machine learning methods in analysing lumbar spine MRI’s has previously shown no consistent advantage for either method [1]. Diagnostic accuracy has been reported for many AI-based diagnostic models; however, their performance across diagnostic tasks and datasets ranges widely [2]. Numerous issues remain to be addressed in this area such as the overwhelming lack of standardization among the datasets and methodologies, which hinders the ability to make comparisons or generalize findings from one study to another [2]. In addition, studies show that performance in an external validation setting often falls short of those achieved via an internal validation setting indicating potential deficiencies in the robustness of the model and how applicable it will be to real-world applications [3]. Some examples of limitations include a heavy reliance on subjective radiologist annotations, lack of recognized diagnostic standards, which causes uncertainty when using the models [3], and many models are created on small or heterogeneous datasets with no external validation, which restricts clinical deployment [1]. Types of CAD methods that have been investigated included semi-automated methods as well as fully automated methods. For example, segmentation-based CAD methods have demonstrated high reproducibility and good agreement between radiologists and their results, but tend to require manual initialization and multiple pre-processing steps, which impede their ability to perform in a fully automated and robust manner when utilized in practice [4]. The examination performed by Goncalves et al. assesses Lee’s classification system for lumbar disc herniation through the employment of sagittal T2-weighted MRI scans derived from 82 cases in a study that involved three orthopaedic specialists of the spine along with one radiologist. The results of the study revealed the extent of inter- and intra-observer reliability ranging from moderate to very high levels [5]. Deep learning (DL) methods often need big labelled datasets, lots of computing power, and complicated tuning, which can prevent use in areas with limited resources. Classical machine learning provides a comparatively simple framework that can be investigated for lumbar MRI classification, particularly when computational efficiency and reproducibility are important considerations when applied with good pre-processing and feature engineering. However, the existing literature has studied either DL or comparatively few have systematically evaluated CML under consistent pre-processing conditions. In response to these gaps, this study presents a structured CML pipeline for the classification of lumbar disc herniation from MRI images. This includes a series of pre-processing techniques (including bilateral smoothing and intensity normalisation) to create features for PCA, which are then used to classify multiple classifiers. Accordingly, the present study focuses on evaluating the performance of a structured CML workflow—without aiming to prove direct equivalence or superiority over deep learning approaches—while yielding good results. Intervertebral discs are flexible structures positioned between vertebrae, which provide mechanical load absorption and permit movement of the spine. Each disc comprises two anatomical components, that is, annulus fibrosus and nucleus pulposus. Where these structures become degenerated and/or under mechanical stress, herniation can occur and potentially compress adjacent nerves as illustrated in Figure 1.

**Figure 2:**
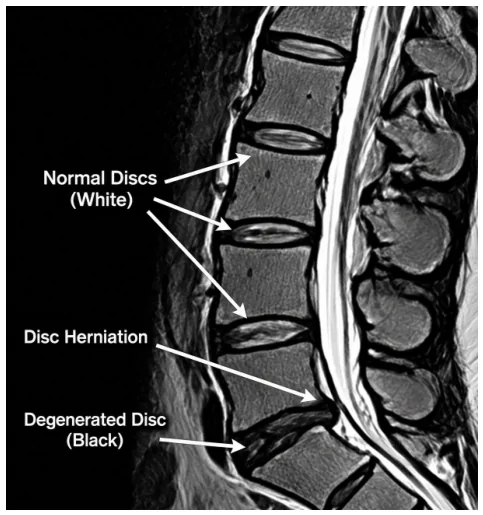
Representative lumbar MRI appearance illustrating normal disc morphology and a herniated disc. Signal-intensity changes may accompany degenerative alterations but are not considered alone as a defining feature of herniation.

Lumbar disc herniation is usually related to pain, neurological symptoms, and functional limitations; incidence is particularly high in the lumbar and lumbosacral regions due to elevated mechanical demands. Timely and precise diagnosis is, therefore, critical if in a given patient, disability is to be reduced, permitting a better outcome. MRI has been used in identifying disc degeneration and herniation but manual interpretation of imaging data takes time and can be subject to inter-observer variability. As seen in Figure 2, MRI can demonstrate morphological changes associated with lumbar disc herniation. However, reduced disc signal intensity may also reflect degenerative changes and should not be considered, by itself, a defining feature of herniation, quite clearly underlining the need for the dependable classification methods.

Research has highlighted a significant research gap that needs to be addressed regarding the effectiveness of well-designed classical machine learning algorithms in providing an optimal solution for lumbar disc herniation classification. Thus, it is necessary to understand if classical machine learning platforms can provide consistent and ideal performance without utilizing deep learning models. This research seeks to examine the effectiveness of a classical machine learning platform for binary classification (normal/abnormal) for lumbo-sacral herniation utilizing medical imaging data. The proposed classical machine learning framework utilizes pre-processing, feature scaling, Principal Component Analysis (PCA) and multiple classifiers integrated into a cohesive and reproducible environment. The following are the primary contributions of this research:

1. To develop a complete and reproducible classical machine learning pipeline for lumbar MRI classification.
2. To perform a systematic evaluation of multiple classifiers under consistent pre-processing protocols.
3. To conduct a study into the effects of bilateral smoothing and PCA on the classification accuracy of the models.
4. To determine the best possible model when developing an automated diagnostic tool within a resource-constrained environment.

The rest of this document will consist of the following. Review of related work through deep learning and classical machine learning methods in Section 2, methodology for the proposed technique, including data acquisition/pre-processing, representation of features through dimensionality reduction, and classifying models in Section 3, experimental arrangements/results including an overall performance analysis in Section 4, comparison of machine learning methods against similar to Section 5, and in Section 6, a full discussion of all results obtained. Finally, Section 7 will present concluding remarks while Section 8 will discuss directions for future work being done in this area.

## 2. Related Work

Recently, there have been more studies looking into how to find automated methods of diagnosing lumbar disc herniation. A variety of methods have been tried to evaluate spinal medical images (especially MRI because of its excellent ability to visualize the intervertebral disc anatomy). Most studies can be defined as either deep learning-based methods or statistical/classical machine learning-based methods. This section reviews some representative studies from each category to help guide the future of research on automated diagnoses of lumbar disc herniation using these methods.

### 2.1. Deep Learning-Based Approaches

Over the past few years, medical image classification diffusion has largely been influenced by deep learning techniques. CNNs have been extensively studied for analysis of lumbar spine MRIs as they allow for automatic extraction of hierarchical features. Many studies have proposed framework architectures of CNNs for the detection and classification of abnormalities in lumbar discs [6,7,8,9,10,11,12,13,14,15,16,17,18,19,20,21,22,23,24]. Tumko et al. (2024) [6] aimed to identify and classify lumbar spinal stenosis in MRI, which includes segmentation, binary classification, and severity grading. The model is performs competitively vs expert radiologists and provides high sensitivity, with high AUROC. The model is dependent on radiologist’s annotations as ground truth, which are time-intensive to complete and may provide inherent bias based upon the amount of manual labelling performed, limiting the model’s scalability. The lack of standardized diagnostic criteria are a barrier to consistent evaluation [6]. Zhang et al. (2023) [7] constructed a deep learning model for auto-detection and classification of lumbar disc herniation using Faster R-CNN (region detection) and ResNeXt101 (classification), with an AUC of 0.90, and very good agreement with expert annotation. However, performance fell off when assessed on external datasets, suggesting challenges of generalization outside of the dataset, as well as being hampered as a result of having to rely on manual annotation and the problem of class imbalance [7]. Bharadwaj et al. (2023) [8] proposed a deep learning approach to automated classification (interpretable) of lumbar spinal pathologies from MRI by integrating V-Net segmentation with BiT-based CNN and decision trees (interpretable); they achieved good performance at AUROC values (0.90) and showed strong agreement with radiologist interpretation results. In this case, the interpretable model achieved better performance than CNNs for multi-class grading (compared to expert review). The authors indicate that the limitations of their work include a relatively small dataset previously collected from a single institution, which reduces the generalizability of the system [8]. Alsmirat et al. (2022) [9] used CNN-based CAD to automatically identify and categorize lumbar disc herniations from MRI using a combination of ROI extraction, data augmentation, and transfer learning. They indicated their model demonstrated overall accuracy of 95.65% for detection and 91.38% for classification, but they had a relatively small dataset (164 images) and manually selected the ROIs, limiting the robustness and generalizability of their results [9]. In the study of Tsai et al. (2021) [10], a deep learning framework was created using YOLOv3 to create an automatic lumbar disc herniation detection system on MR images. The authors used both data augmentation and the standard conditions of the YOLOv3 network, achieving an average mAP of 92.4%. However, the authors utilized a small, imbalanced sample for the study; therefore, the sample size will limit the generalizability of the results [10]. Prisilla et al. (2023) [11] developed a DL framework to automatically detect lumbar disc herniation in magnetic resonance imaging (MRI) via YOLOv5, YOLOv6, and YOLOv7; YOLOv5x produced the most precise detections (89.3% mean average precision) of all three models considered. Therefore, their work only focuses on detection and does not include any forms of classification or degree of herniation; subsequent to this, their work could not be implemented or used in a clinical environment [11]. Yilihamu et al. (2025) [12] proposed a multi-phase pipeline employing YOLOv8 to automatically quantify (by assessing canal area in square mm) and classify lumbar disc herniation using both detection and segmentation models as well as keypoint models. A large multi-institutional image dataset of 25,000 images was used to train the proposed system, achieving a high performance (IoU = 97%) as well as consistent classification when tested on multiple datasets. However, the complex structural architecture of the framework and the need for large amounts of annotated data may limit its scalability and ability for real-time deployment in a clinical setting [12]. Zhou et al. (2019) [13] proposed a similarity-based CNN for the automated detection of lumbar vertebrae on MRI scan images. The authors demonstrated a high level of accuracy by achieving a localization rate of 98.6% while using incomplete annotation information. A limitation of this method is its reliance on a similarity-based matching function and therefore is not likely to produce a high level of precision in more complex/noisy scenarios [13]. Pan et al. (2025) [14] also proposed a deep learning framework for classifying lumbar disc herniation by using Nearest Neighbour dual-path contrastive learning, which provides a way to integrate global and local features. The performance of this approach was superior to that of conventional CNNs and it provided better generalization; however, the computational complexity of this approach limits its practical application [14]. CMMF-Net was introduced by Lu et al. (2023) [15] to be a collaborative deep learning solution that integrates feature selection network with a classification network utilizing ResNet-18 in three dimensions. In this process, the model in their work combined cropped images, features on various scales, and segmentation-derived distance so as to provide a classification accuracy of 91.32% and Dice coefficient of 94.39%. Nevertheless, pixel-level annotation was needed in the process and end-to-end training was not realized [15]. A CNN-based method developed by Wan et al. (2025) [16] implemented V-Net for reconstruction of axial lumbar disc CT and U-Net for both disc segmentation and identification of multi-scale unit. Its results, based on the input of 440 patients, showed that the Dice coefficient equalled to 0.926 in segmenting lumbar disc, whereas radiologist assisted by AI has an accuracy of examination equal 92–95%. Nevertheless, this framework is designed for performing multi-class evaluation on CT images, rather than binary evaluation using MRI [16]. The study of Al-Kubaisi and Khamis (2022) [17] investigated the use of transfer learning for lumbar disc conditions according to axial T2-weighted MRI scans and the proposed ROI extraction technique. Their dataset comprised 905 disc images which were labelled as normal or abnormal, where the abnormal class was composed of degeneration, bulging, and herniation. A same-domain pretrained model using unlabelled MRI images and the VGG16 deep learning model boosted the accuracy to 89.01% and yielded an F1-score of 90.91% [17]. The authors Wang et al. (2025) [18] built up a new version of YOLOv5 model that allows the diagnosis of disc herniation and degeneration grades based on the T2-weighted MRI received from the axial and sagittal views. The model was tested on 420 patients at the facility and on 52 external patients and achieved an F1-score of 0.91 for herniation detection in the internal testing in axial and sagittal approaches while the external F1-scores constituted 0.87 and 0.91, respectively [18]. A study by Su et al. (2022) proposed the development of a ResNet-50 architecture in a multitasking neural network for the detection of lumbar disc herniation, central spinal stenosis, and nerve root compression, based on T2-weighted axial MRI images 15,254 internal and 1273 external images were used for the purpose, achieving lumbar disc herniation accuracy results of 84.17% and 74.16% respectively, with AUC of binary grading equalling to 0.97 and 0.95 respectively [19]. Zhang et al. (2024) [20] developed an MRI-guided automated decision-support system for completely automated diagnosis of lumbar disc herniation, including its localization, MSU classification, and Pfirrmann grading. The results obtained by using the system on 3255 lumbar discs from 217 patients demonstrated accuracy rates of 95.83% for herniation diagnosis, 95.0% for MSU classification, and 83.5% for Pfirrmann grading [20]. Tan et al. (2024) [21] introduced the two-part deep learning model for identifying lumbar discs and for classifying the herniated and healthy discs using sagittal T2-weighted MRI scans. The NLIC model for the experimental setting consisted of 160 discs from 32 individuals and earned an accuracy of 93.75% with F1-score of 92.27% from the original unbalanced dataset and later on the augmented dataset showed values of 96.88% and 96.46% respectively [21]. Liu et al. (2024) [22] described comparisons of ResNet-34, DenseNet-121, and MobileViT for the identification of calcified and non-calcified cases of lumbar disc herniation assessed on sagittal MRIs. In a multicentre cohort of 1224 subjects, ResNet-34 had an accuracy of 91.67% with AUC of 0.96 on the internal testing dataset with respective external validation values of 88.76% and 0.88 [22]. BianqueNet was developed by Zheng et al. (2022) [23] to help segment and quantitatively assess lumbar intervertebral disc degeneration using T2-weighted magnetic resonance imaging in the mid-sagittal plane. The model achieved a DICE score of 94.80% for intervertebral discs. Quantitative classification was also assessed using overall F1-scores, achieving 92.02% and 90.63% for two datasets, respectively. However, this framework was used to evaluate intervertebral disc degeneration rather than to classify herniated and normal intervertebral discs [23]. GE-YOLOv8 was developed by Guo et al. (2025) [24] for detection and MSU grading of lumbar disc herniation from the axial T2-weighted MRI images. The model achieved mAP50 of 78.0% for internal validation and 62.9% for external validation, while mAP50–95 was calculated for 67.1% and 52.3%. Nevertheless, this framework performed multi-class localization and grading of herniation, but did not perform binary classification using sagittal MRI [24]. The vast majority of recent studies on the detection and classification of lumbar discs use deep learning, primarily CNNs, and also leverage end-to-end CNN-based methods for automatic feature extraction from MRI data. These methods are not only able to achieve exceptional performance; they require substantial amounts of annotated data, are computationally expensive and involve multiple tuning parameters, and due to the limited interpretability of CNNs, decrease transparency of the model for clinical decision-making and ultimately decrease the practical application. As a result, traditional ML methods have also been utilized to detect and classify lumbar disc abnormalities.

### 2.2. Classical Machine Learning-Based Approaches

Apart from deep learning-based approaches, traditional machine learning methods have been used to classify lumbar disc herniations by using handcrafted features together with classifiers (i.e., SVM, kNN, Random Forests). Despite providing lower costs and supporting greater interpretability than their deep learning counterparts, less research has been conducted on these methods [25,26,27,28,29,30,31,32,33,34,35,36,37,38]. Jafari et al. (2019) [25] compared classical techniques (kNN, SVM, and logistic regression) in classifying lumbar disc herniation using non-MRI clinical data, and they found that SVM showed superior performance on small datasets while kNN performed better on large datasets, indicating a significant effect of dataset size on performance. However, there are limitations imposed on diagnostic accuracy due to the reliance on clinical data that does not involve images [25]. Ghosh et al. (2011) [26] proposed a classical CAD system for diagnosing lumbar disc herniation from MRI using handcrafted features (LBP, Gabor, GLCM, shape, intensity), followed by PCA/LDA and classification with kNN, SVM, and Naïve Bayes. The method achieved high performance (accuracy up to 98.29%, sensitivity 98.11%). However, it was evaluated on a very small dataset (35 cases) and relies on extensive feature engineering, limiting scalability and robustness [26]. Ebrahimzadeh et al. (2018) [27] proposed a machine learning-based CAD system for diagnosing lumbar disc herniation from MRI using intensity and shape features from segmented discs, combined with classifiers such as SVM, kNN, and MLP. The method achieved high performance (SVM accuracy up to 97.8%) with relatively low computational complexity. However, it was evaluated on a small dataset (30 cases) and relies on multiple pre-processing and feature engineering steps, limiting robustness and generalizability [27]. Tan et al. (2018) [28] proposed an NLP framework for extracting findings related to the lumbar spine from radiology reports. They also compared the use of rule-based and machine learning methods. The performance of all of the machine learning methods was superior to the rule-based methods in terms of sensitivity (0.94 versus 0.83, AUC 0.98 versus 0.90) with similar specificity but was dependent on the quality of the radiology report and required annotated data, and also did not analyse the images directly; thus, they have limited diagnostic capabilities [28]. Oktay and Akgul (2011) developed a machine learning method for automatically localizing lumbar discs in MRI by using probabilistic graphical models and SVMs to develop features using PHOG; this method achieved 95% accuracy; however, it extends only to localization (not classification) and primarily relies on handcrafted features and was implemented as a multi-stage approach, which limits its ability to scale [29]. Chiu et al. (2023) also developed a machine learning method to predict clinical outcomes after lumbar nucleoplasty, using MRI-derived radiomics features as well as clinical features to develop multiple prediction models; the best model was developed using improved Random Forest and achieved an accuracy of 0.76 and an AUC of 0.77; however, the target of this is prognosis rather than diagnosis and is developed using a limited dataset, which negatively affects the generalizability of this method [30]. Kocaman et al. (2023) evaluated multiple machine learning methods for predicting lumbar disc herniation, using anatomy derived from MRI, and found that XGBoost performed the best with an accuracy of 92% and AUC of 0.94; however, this was accomplished using manually extracted features and a single-centre dataset, which limits generalizability [31]. Ghosh et al. (2012) [32] proposed a machine learning method for automatic localization of lumbar discs in MRI using HOG features with an SVM classifier and heuristic rules, achieving high accuracy (≈99%). However, it focuses on localization rather than classification and relies on handcrafted features and multi-stage processing, limiting scalability [32]. Lin et al. (2024) [33] proposed a machine learning framework to predict lumbar disc herniation from X-ray-derived disc height features using models such as LASSO, Random Forest, and XGBoost. While these features were informative, performance remained moderate (F1 = 0.78). However, reliance on handcrafted features and the absence of deep image-based learning may limit accuracy and generalization [33]. Athertya et al. (2019) [34] proposed a machine learning approach for detecting Modic changes in spinal MRI using LBP-based texture features with classifiers such as SVM, kNN, and Random Forest, achieving up to 91.7% accuracy with data augmentation. However, reliance on semi-automatic segmentation and handcrafted features may limit robustness and scalability [34]. Han et al. (2022) [35] examined ML/deep learning techniques [cnn] for lumbar disc mri images and compared the model’s results with human observations. The model produced performance results similar to expert observers via roc analysis. The provided model focuses on statistically comparing results and does not provide complete diagnostic modelling, limiting application to fully automated clinical systems [35]. Chen et al. (2025) [36] developed ml models to predict recurrence rates of lumbar disc herniations following endoscopic discectomies using both clinical and imaging data from 309 patients. Random Forest and XGBoost performed best, providing key predictors of recurrence—bmi, pdhi, spinal canal stenosis—while limiting findings to prognostic and non-imaging-based diagnosis using structured features, thus limiting application to fully automated mri classification [36]. According to Tang et al. (2025) [37], a classification system has been developed that uses a sagittal MRI approach for diagnosing lumbar disc herniation on the basis of destruction of the endplate. XGBoost, LASSO, and bootstrap logistic regression were used to analyse the variables present in the system, which relate to the necessity of surgical intervention. Their findings suggested that age and type of classification were the significant variables as they were retained in the form of a nomogram with a respective AUC of 0.74 from the validation of 50 patients. Nevertheless, their model seems to explain the treatment choice in terms of observational variables instead of directly classifying the MRI [37]. In this sense, Saravi et al. (2023) [38] combined preoperative T2-weighted MRI data with clinical data for predicting specific outcomes after surgery in lumbar disc herniation patients utilizing specific machine learning and neural network classification systems. The results revealed that the used combination of variables enabled achieving the overall mean accuracy index on the dataset of 88.17%, compared to 87.69% in the case of using clinical variables alone. Despite that, the research dealt with predicting the postoperative outcomes and did not aim to directly distinguish between normal and herniated discs [38]. When comparing traditional machine learning approaches to deep learning, traditional approaches tend to utilize handcrafted features (pre-processed) with classifiers such as SVMs and KNNs. Classical machine learning and deep learning studies differ substantially in their datasets, feature representations, tasks, and evaluation protocols. Therefore, reported performance values across these studies should be interpreted descriptively and should not be considered evidence of direct performance equivalence or superiority between approaches. Thus, making direct comparisons and evaluations between studies is difficult due to differing evaluation protocols, representation of features, and experimental environments.

### 2.3. Research Gap

There has been substantial development made in automated detection of lumbar disc herniation but many limitations still exist. Most of the recently published research papers were done with deep learning architectures that require both extensive amounts of annotated data as well as a lot of computational power. Despite their high performance, such complexity limits practical deployment in resource-constrained settings. In contrast, classical machine learning approaches have received less attention, despite advantages in computational efficiency, interpretability, and simplicity. Existing ML studies also vary in pre-processing, feature representation, and evaluation protocols, hindering direct comparison. Therefore, a systematic investigation of classical machine learning pipelines under a unified and reproducible framework is needed. This study addresses this gap by evaluating a structured ML pipeline using consistent pre-processing, dimensionality reduction, and evaluation strategies.

## 3. Methodology

### 3.1. Overall System Pipeline

**Figure 3:**
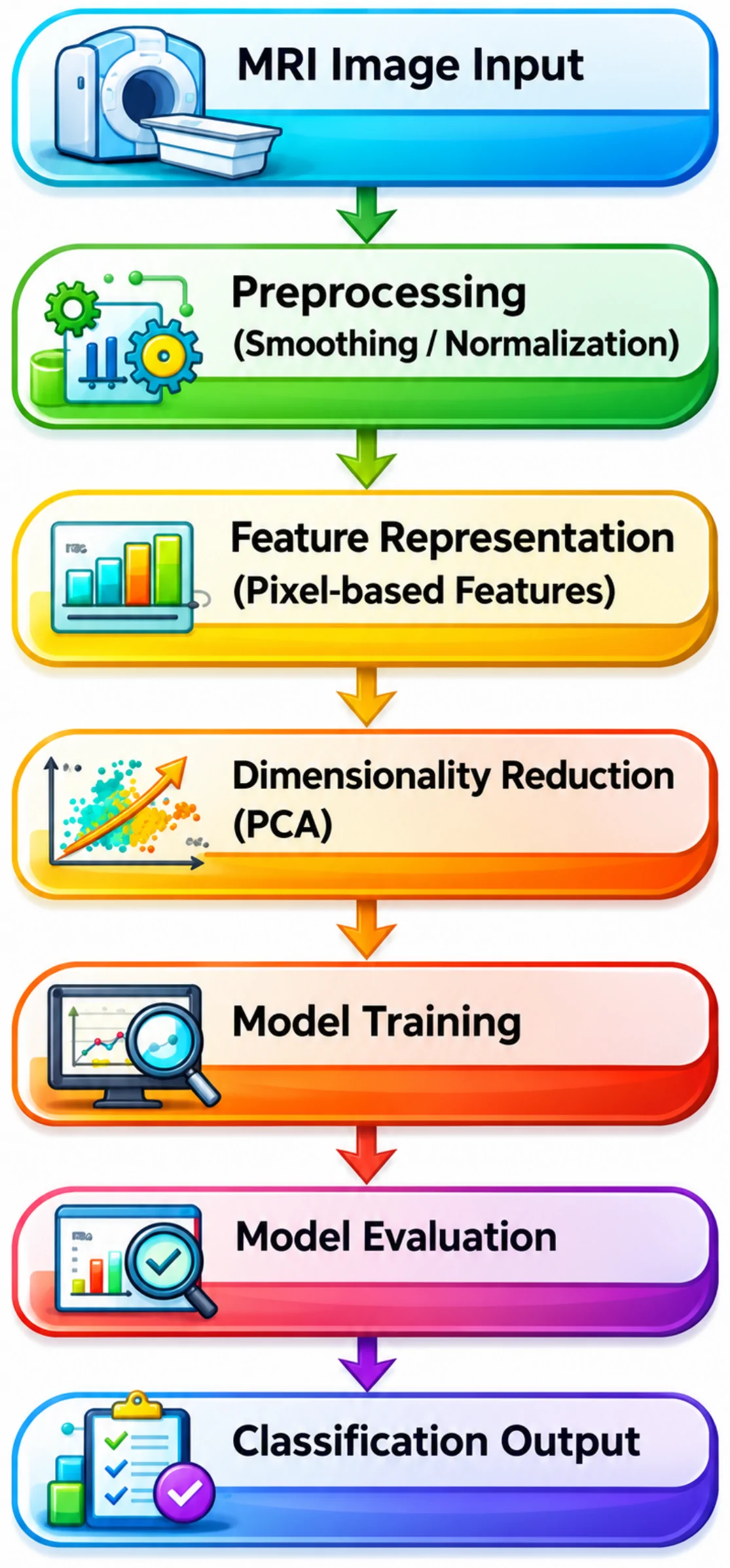
Overview of the proposed lumbar disc classification pipeline, including pre-processing, feature representation, PCA-based dimensionality reduction, and model training and evaluation.

Lumbar intervertebral disc (IVD) condition (normal vs. herniated) classification is performed on MRI images via a systematic reproducible workflow. Each input represents a sagittal lumbar MRI image, and the task is formulated as binary image-level classification into normal or herniated. The current framework does not perform disc-level localization, anatomical-level classification, severity grading, or estimation of the number of affected discs. The workflow is accomplished through five steps: first, by acquiring the images; second, by pre-processing the images to reduce variability between them (by smoothing, normalizing, and standardizing the images); third, by converting each image into a pixel-based numeric representation of the features in order to prepare for machine learning; fourth, by retaining the most significant variance contained in the feature space via Principal Components Analysis (PCA); and fifth, by training and testing several traditional classifiers such as support vector machines (SVMs), k-Nearest Neighbour (kNN), Random Forest (RF), and XGBoost algorithms on the newly created features to perform IVD classification predicted by the trained models. The complete workflow of the steps described above can be found in Figure 3.

### 3.2. Data Acquisition and Exploratory Analysis

The dataset consisted of 1426 sagittal lumbar MRI images collected from multiple sources. All included images were sagittal T2-weighted lumbar spine MRI images. Scanner manufacturer/model, field strength, and detailed acquisition parameters (e.g., repetition time, echo time, slice thickness, and pixel spacing) were unavailable in the assembled dataset. These metadata were not model inputs; the framework operated solely on image-pixel information. Their absence therefore precludes scanner-stratified analysis, but not evaluation of the stated exploratory image-level objective. Of these, 368 images were obtained from King Salman Specialist Hospital in Ha’il, Saudi Arabia, and 258 images were obtained from King Khalid Hospital in Ha’il, Saudi Arabia. The remaining 800 images were obtained from publicly available sources, including Kaggle. Clinical practices that widely utilize MRI benefit from its capacity to provide high-quality imaging of soft tissue, spinal anatomy, and the morphology of intervertebral discs, which make it a desirable modality to detect abnormalities to the lumbar discs (e.g., degeneration and herniation). This study collected and organized a total of 1426 MRI images into two classes based on the clinical condition of the intervertebral discs: 671 images correspond to the class of normal intervertebral discs, and 755 images correspond to the herniated disc class. All of the MRI images have been acquired in the sagittal view. Images were included if they were sagittal lumbar MRI scans suitable for radiological assessment and could be confidently classified as either normal or herniated. Images acquired in views other than sagittal, as well as cases with unclear or difficult-to-interpret radiological findings that could not be confidently assigned to either class, were excluded from the final dataset, which provides a consistent and valuable representation of the intervertebral disc anatomical structures, supporting reliable identification of any abnormalities in the intervertebral disc. Prior to developing any predictive models, exploratory data analysis (EDA) was performed to characterize and describe the dataset structure and distribution of MRI images. The EDA entailed visually inspecting samples representative of both classes of intervertebral discs to observe differences in morphology and related patterns of signal intensity so as to understand the fundamental structure of the dataset and to inform the design of the subsequent pre-processing and features extraction steps. A summary of the distribution of MRI images according to class can be found in Table 1.

**Figure 4:**
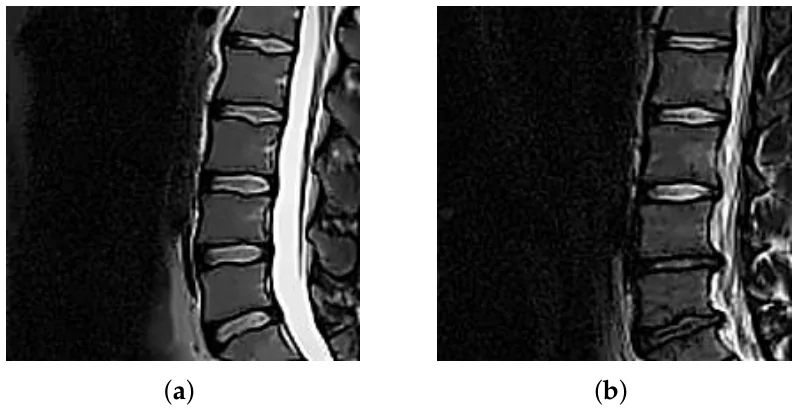
Representative sagittal lumbar MRI images showing (**a**) a normal disc and (**b**) a herniated disc with structural deformation and reduced signal intensity.

Figure 4 presents pictures of the MRI data for both classes used to identify specific characteristics visually before undertaking to assess the images for automation. In general, normal intervertebral discs have a uniform structure compared with herniated intervertebral discs, which have undergone morphological deformation and show lower levels of signal intensity than normal discs. Both signal patterns and morphological characteristics provide a basis for visually distinguishing between normal and herniated discs. In addition, these same differences provide a basis for automated classification systems.

**Figure 5:**
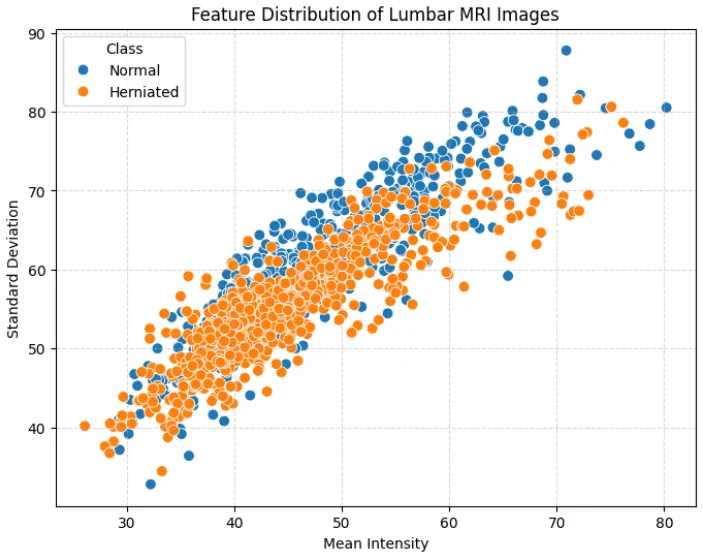
Feature distribution showing mean intensity versus standard deviation for normal and herniated MRI images, with each point representing one image.

To examine the statistical characteristics of the data, global intensity characteristics (mean pixel intensity) and structural variations (standard deviation) of each MRI were evaluated. The distributions of the pixel intensities for normal and herniated IVDs were revealing structured patterns with observable trends across the two groups as shown in Figure 5. Although there is some overlap, the two distributions have distinct regions that indicate meaningful statistical differences between classes. Therefore, based on these results, pixel intensity-based feature vectors can provide useful discriminative information, while the relationship between mean pixel intensity and standard deviation demonstrates the applicability of dimensionality reduction techniques in the next steps.

**Figure 6:**
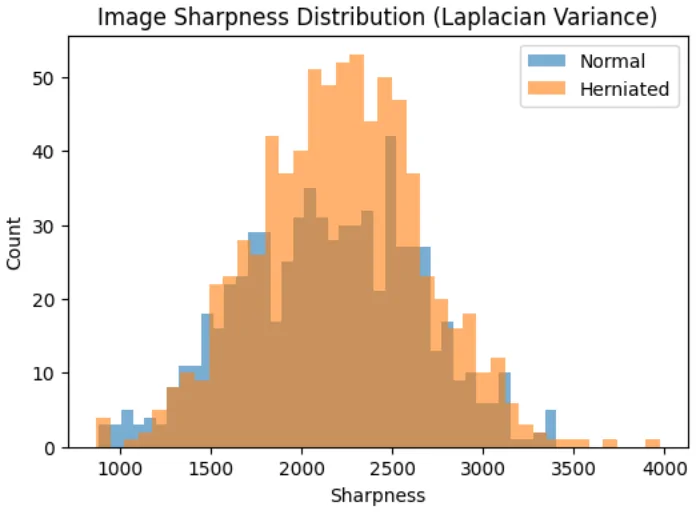
Distribution of image sharpness (Laplacian variance) for normal and herniated lumbar MRI images.

In addition, image sharpness quantification through the variance of the Laplacian informs the edges and fine detail in each MRI image can provide insight into the structural differences. In Figure 6, the variance in sharpness values reveals large differences in the structural complexities of the normal and herniated samples with similar variations within classes. The large degree of variability indicates that noise and fine detail are likely to exist within the image and contribute to the lack of stability in feature sets and consequently justifies the need for the application of noise reduction techniques (e.g., smoothing) that significantly minimize irrelevant high frequency variations while retaining the critical structural characteristics.

**Figure 7:**
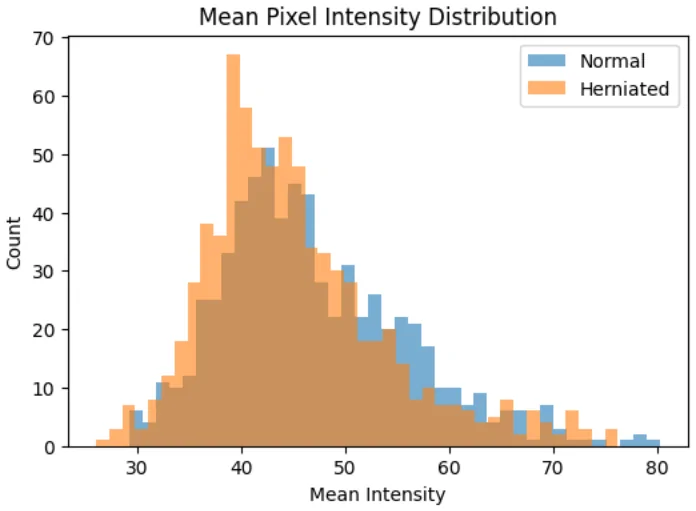
Distribution of mean pixel intensity for normal and herniated lumbar MRI images.

Mean pixel intensity is a descriptive statistic for average signal levels and overall brightness levels in MRI images. As illustrated in Figure 7, normal and herniated samples exhibit a high degree of overlapping intensity ranges, indicating very similar global characteristics. However, the lower intensity ranges are represented by a higher frequency of herniated samples and the mid and higher intensity ranges by normal samples, thus indicating that herniated samples tend to be darker relative to other structural characteristics.

### 3.3. Data Pre-Processing

Variations in spatial resolution, noise characteristics, and intensity distributions within and between medical images comprise an established concern. These variations can lead not only to misclassification of medical images but also to reduced model stability and generalization performance if they are not properly addressed. A structured pre-processing pipeline was utilized to standardized MRI images, thereby providing a common basis on which to assess MRI data. The pre-processing approach included four elements: image resizing to provide a consistent spatial resolution; noise reduction via smoothing to reduce high-frequency variations; intensity normalization to reduce inter-scan differences; and feature standardization to ensure a consistent scaling of features across all images. As a result, these four processes provided a consistent and stable input representation to allow further feature extraction, dimensionality reduction by Principal Component Analysis (PCA), and classification.

#### 3.3.1. Image Resizing

**Figure 8:**
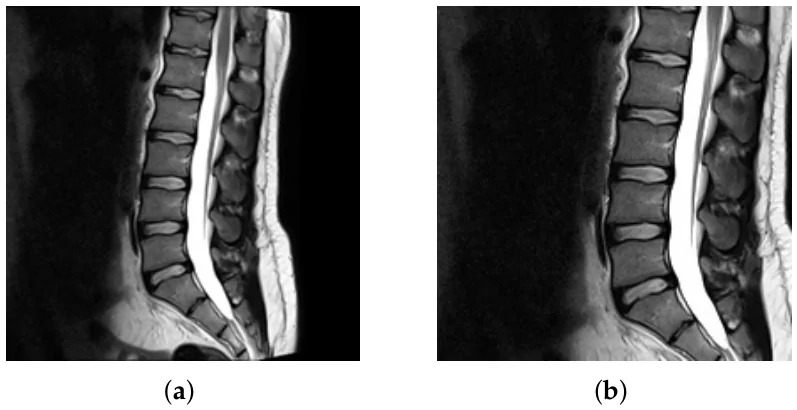
Image resizing process: (**a**) original lumbar MRI image and (**b**) resized image (200 × 200 pixels).

MRI scans collected from different sources have large variations between their spatial resolutions and how they are sized, which makes the process of feature extraction and training models difficult. Hence, every scan was resized to 200 × 200 pixel resolution. This allows all images to have the same spatial representation within the dataset (uniform standardization of size) and eases feature representation and therefore reduces computational complexity while maintaining all important aspects of anatomy necessary for classification. Figure 8 shows how the resized image is a standard form of the original MRI scan, while still keeping the overall structure of the spine, just changing the size.

#### 3.3.2. Noise Reduction (Smoothing)

**Figure 9:**
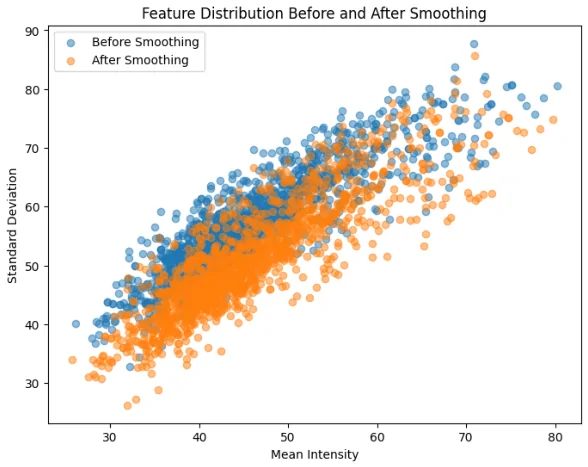
Feature distribution before and after smoothing, based on mean intensity and standard deviation for each MRI image.

Some scanning systems such as MRI are strongly influenced in their ability to provide accurate visual data as influenced by noise and small fluctuations in signal intensity. Before modelling, noise and small intensity fluctuations are diminished by using a smoothing process for high-frequency noise while preserving all anatomical features in the MRI images. Figure 9 shows that as the data is smoothed, the distribution of features forms a more compact and less dispersed distribution than the originally identified feature distribution. While the relationship between mean intensity and standard deviation remains the same, the closer location of the data points represents less variability due to noise and provides a more stable feature space that logically supports consistent features for more accurate model training.

**Figure 10:**
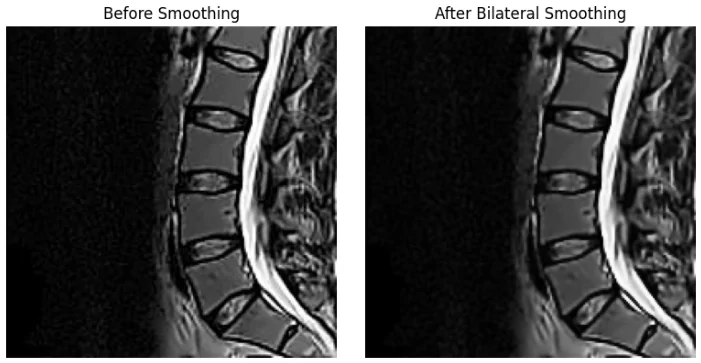
Before and after smoothing images: on the (**left**) is the original image and on the (**right**) is the smoothed image with reduced noise and improved intensity uniformity.

As a visual comparison, Figure 10 shows that the original image has noticeable high-frequency noise, as well as small fluctuations in intensity that create slight distortion to subtle structural details of the lumbar spine. When compared side-by-side with the smoothed image, it is clear that the structural features of the lumbar spine (e.g., intervertebral discs and the associated surrounding tissues) are preserved but with less noise and with smoother transitions in intensity throughout the surrounding anatomical structures. The smoothing process effectively removes nonessential variation while retaining important structural elements necessary to make a thorough analysis.

#### 3.3.3. Intensity Normalization

**Figure 11:**
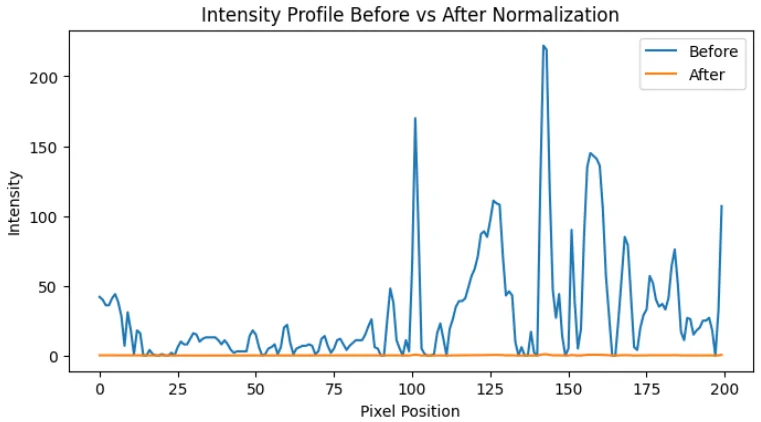
Pixel intensity profiles before and after normalization, showing reduced variability within a consistent intensity range.

Different MRI sources can produce varying intensities based on acquisition conditions, leading to inconsistency of features and models. The intensity was normalised so that pixel intensity is the same within the same level of intensity. Normalised intensity is more valuable to the learning algorithms that rely on scaling features (for example, kNN and SVM) and also provide better results for dimension reduction techniques such as PCA. Normalising image intensity is illustrated in Figure 11, which illustrates that after normalisation, the level of intensity variation has decreased and greater reliability exists in the structure of the overall image.

#### 3.3.4. Image Standardization

**Figure 12:**
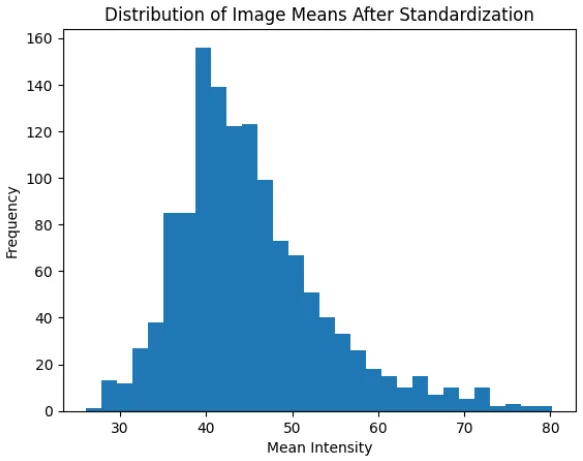
Distribution of image-level mean intensity after feature standardization.

Following the normalisation of the intensity of the image, feature standardisation was implemented using z-score standardisation (StandardScaler), in order to have a mean of zero and a standard deviation of one for every pixel. Combining the pixel values (intensities) of the features into a model ensures that they will not be weighted differently due to their respective ranges within the overall dataset. This may affect the learning algorithms based on distance and margin factors (kNN and SVM). As illustrated in Figure 12, standardising features has created a narrower distribution with less spread, indicating that there is less variability in the average intensity for the pixels and hence a more reliable space for creating a classifier.

**Figure 13:**
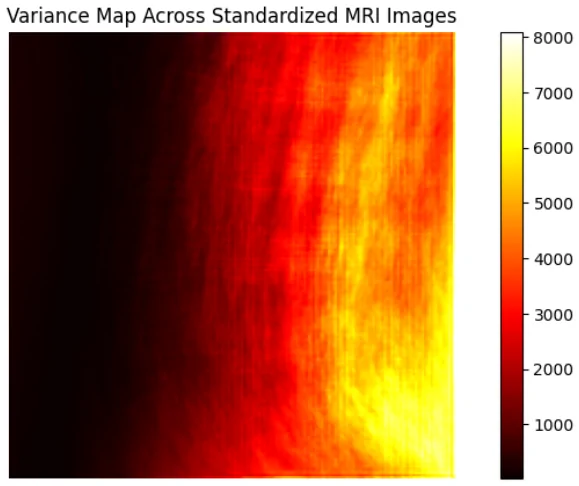
Variance map across standardized lumbar MRI images, highlighting pixel-wise variability.

To quantify the amount of spatial coherence in the spatial distribution across feature-standardised MRI images, a variance map was created to depict the spatially distributed variance across the images. As shown in Figure 13, variance was found to be distributed non-uniformly, with regions that are anatomically important being associated with high variability and homogeneous areas associated with low levels of variability. These results indicate that when standardising features, meaningful structural differences are preserved and irrelevant intensity variations are reduced, supporting consistent feature extraction and creation of reliable classifiers.

### 3.4. Feature Representation

**Figure 14:**
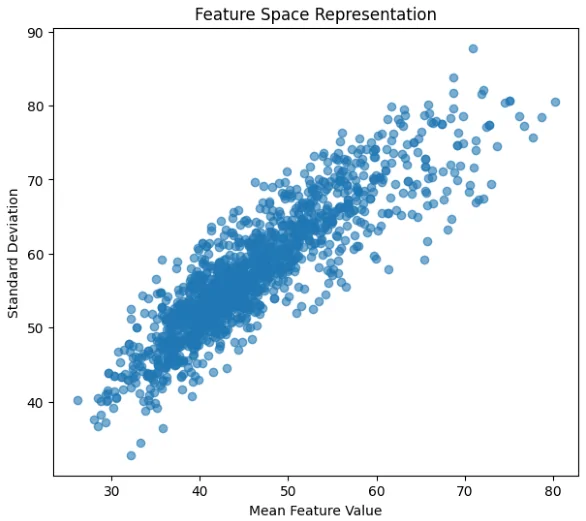
Feature space representation of pixel-based MRI features, showing mean values versus standard deviation.

MRI images were converted to numerical feature vectors through pre-processing (pre-processing) of 200×200 images into one dimension (40,000), retaining pixel intensity. A review of the distribution of the features (Figure 14) showed that the relationship of the mean versus standard deviation produces a structured result, meaning the pixel-based representation was able to effectively capture differences in mean intensity and texture. Additionally, the feature space is very high-dimensional, therefore increasing the complexity of calculating the values, which can negatively impact the performance of the analysis, leading to the need to decrease dimensions through Principal Component Analysis (PCA).

**Figure 15:**
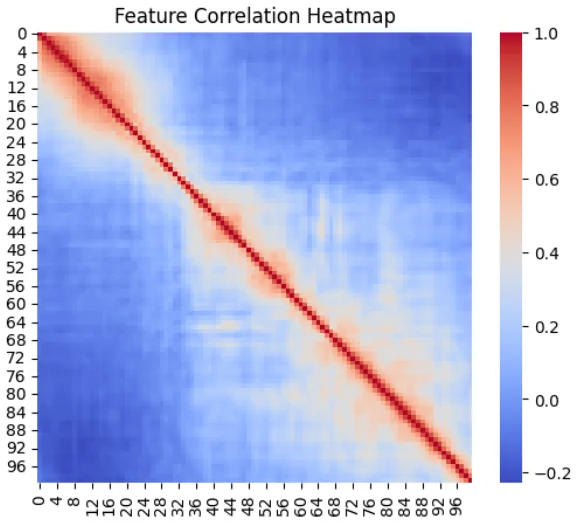
Feature correlation heatmap for a subset of extracted features, showing inter-feature dependencies.

Feature relationships were assessed by performing correlation analysis in a slice of the feature space. The heatmap in Figure 15 shows a high correlation, particularly along the diagonal, indicative of considerable redundancy among features and could also indicate structured dependencies outside the diagonal. The results of this analysis indicate that the pixel-based representation contains redundant information; therefore, dimensionality reduction methods (e.g., PCA) will be required to retain the information in the useful aspects of the features and improve the efficiency of computations.

### 3.5. Dimensionality Reduction Using PCA

**Figure 16:**
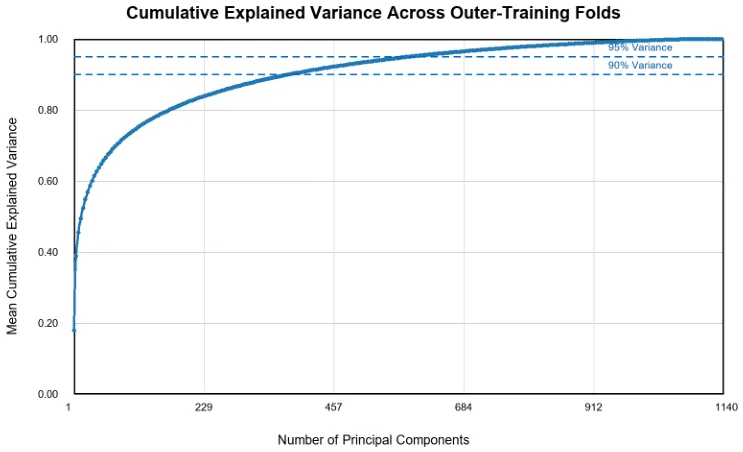
Mean cumulative explained variance versus the number of principal components across the five outer-training partitions.

To improve performance and efficiency, the data was reduced due to the large number of features per image (40,000). The complexity of high-dimensional spaces leads to an increased likelihood of overfitting and decreased generalization. PCA was used to project the data onto independent orthogonal components while preserving the maximum amount of variation in the dataset. To prevent information leakage, feature standardization and PCA were fitted only on the training partition within each cross-validation fold. Figure 16 shows the mean cumulative explained variance curve computed from the five outer-training partitions. Across these partitions, approximately 380 components were required to explain 90% of the variance and approximately 592 components were required to explain 95%, demonstrating that the original 40,000-dimensional pixel representation could be substantially reduced.

The number of retained components was treated as a model-selection parameter rather than being chosen from the held-out outer test folds. For each outer fold, candidate values of 10, 20, 40, 60, 80, and 100 components were evaluated using three-fold inner cross-validation. Because Extra Trees achieved the highest final F1-score and ROC-AUC among the evaluated classifiers, Table 2 reports its mean inner-validation accuracy and F1-score, averaged across the five outer folds. The highest mean inner-validation accuracy was obtained with 40 components, whereas the highest mean inner-validation F1-score was obtained with 80 components.

**Figure 17:**
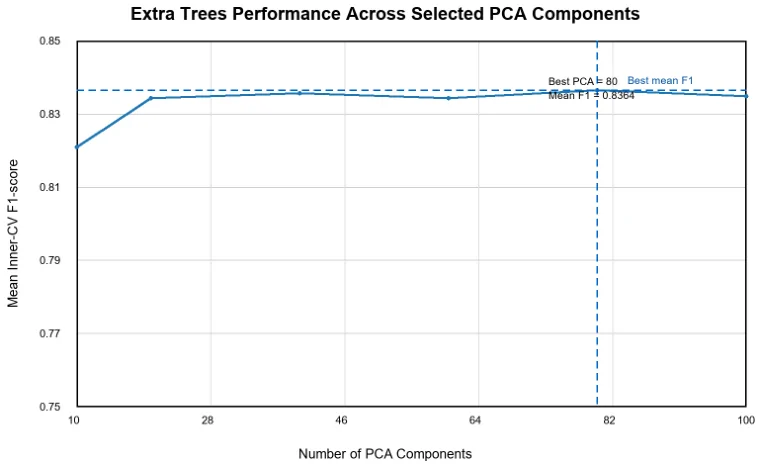
Mean inner-validation F1-score of the Extra Trees classifier across selected PCA component values.

As shown in Figure 17, the mean inner-validation F1-score for Extra Trees increased from 0.8208 with 10 components to 0.8357 with 40 components, changed only slightly at 60 components, and reached its maximum of 0.8364 with 80 components before decreasing to 0.8348 with 100 components. Accordingly, 80 components produced the highest aggregated mean inner-validation F1-score for Extra Trees in this analysis; however, component selection was repeated independently within each outer fold for the final nested cross-validation evaluation. Additional fold-specific PCA-selection frequencies and explained-variance thresholds are provided in Tables S1 and S2 of the Supplementary Materials.

### 3.6. Classification Models

A set of classical machine learning algorithms is used for classification, to rigorously assess the proposed dimensionality reduction and feature extraction pipeline. We consider several models from multiple paradigms, including instance-based learning, linear classifiers, and kernel-based approaches and ensembles, allowing us to assess their performance under different modelling assumptions and decision mechanisms. They are chosen for their ability to work with high-dimensional data and for their strengths in capturing different decision boundaries. Approaches relying on distance, like k-Nearest Neighbors (kNN), utilize local similarity structures while support vector machines (SVMs) build separating hyperplanes to achieve maximum class margins. Ensemble methods like Random Forest, Gradient Boosting, and XGBoost combine multiple weak learners to improve robustness and non-linear relationship modelling. All models were trained and evaluated by using reduced feature space obtained from PCA and fed into the classifier. The framework allows for a fair comparison of the effects of pre-processing and dimensionality reduction against the classification algorithms. Table 3 provides a summary of the models and their respective categories.

The hyperparameters of all classifiers were predefined and kept fixed throughout the comparative experiments. No automated hyperparameter optimization procedure, such as grid search or randomized search, was applied. Parameters not explicitly specified were retained at their corresponding library default values. The complete model configurations are summarized in Table 4.

### 3.7. Evaluation Strategy

Final model evaluation used stratified nested cross-validation at the image level, with five outer folds for performance estimation and three inner folds for selection of the number of PCA components from 10, 20, 40, 60, 80, and 100. Within every inner and outer fold, StandardScaler and PCA were fitted only on the corresponding training partition and then applied to the held-out partition. All ten classifiers were evaluated using the same outer folds. Out-of-fold predictions for all 1426 images were aggregated to calculate accuracy, precision, sensitivity (recall), specificity, F1-score, and ROC-AUC. Ninety-five percent confidence intervals were estimated using 2000 stratified bootstrap resamples of the out-of-fold predictions. Because consistent patient identifiers were unavailable across the combined dataset, patient-level partitioning could not be performed or verified; therefore, possible overlap of images from the same patient across folds cannot be ruled out. Table 5 summarizes the dataset composition and nested cross-validation design used for the final analysis.

## 4. Experiments and Results

This section evaluates the framework for lumbar disc herniation classification from MRI images proposed in this study. The experiments evaluate the effect of pre-processing, PCA-based dimensionality reduction, and a variety of classical machine learning algorithms in a common setting. The complete pre-processing pipeline was applied on the similar features in order for the models to be trained and tested. So, the final results reflect the outcome of the entire proposed methodology. Classifier performance was assessed using accuracy, precision, sensitivity (recall), specificity, F1-score, and ROC-AUC, together with 95% confidence intervals derived from 2000 stratified bootstrap resamples of the aggregated out-of-fold predictions. The next sub-sections give a detailed comparison of models performance and analyse the effect of pre-processing and PCA on classification.

### 4.1. Model Performance Comparison

The ten classifiers were evaluated under the same five outer folds of the nested cross-validation protocol. As shown in Table 6, Random Forest achieved the highest accuracy (0.8436; 95% CI: 0.8240–0.8626), whereas Extra Trees achieved the highest F1-score (0.8586; 95% CI: 0.8416–0.8744) and ROC-AUC (0.9096; 95% CI: 0.8927–0.9254). Extra Trees also achieved a sensitivity of 0.9007 and a specificity of 0.7779. The performance of Random Forest was comparable, with an F1-score of 0.8575 and ROC-AUC of 0.9034, while KNN achieved the highest specificity of 0.8033. AdaBoost produced very high sensitivity (0.9960) but low specificity (0.1580), indicating a strong tendency to predict the herniated class.

### 4.2. Detailed Analysis of Best Model

Extra Trees was selected for detailed analysis because it achieved the highest F1-score and ROC-AUC. As shown in Table 7, the model achieved a herniated-class recall of 0.9007 and F1-score of 0.8586. For the normal class, precision was 0.8744, recall was 0.7779 and F1-score was 0.8233. Thus, Extra Trees detected most herniated images, although its lower recall for the normal class reflects a greater number of normal images classified as herniated. The macro-average F1-score was 0.8410.

### 4.3. Impact of Smoothing

The effect of bilateral smoothing was evaluated by repeating the same nested cross-validation procedure with and without smoothing. Table 8 reports the change in out-of-fold F1-score and the 95% bootstrap confidence interval for the paired difference. Random Forest showed the largest supported improvement, increasing from 0.8410 to 0.8575 (difference: 0.0165; 95% CI: 0.0037–0.0294). AdaBoost also showed a small supported increase of 0.0044 (95% CI: 0.0015–0.0079). For the remaining classifiers, the confidence intervals included zero, providing no clear evidence of improvement or deterioration due to smoothing. Extra Trees was essentially unchanged, whereas the observed decrease for LightGBM was not conclusive because its confidence interval also included zero.

### 4.4. Error Analysis via Confusion Matrices

**Figure 18:**
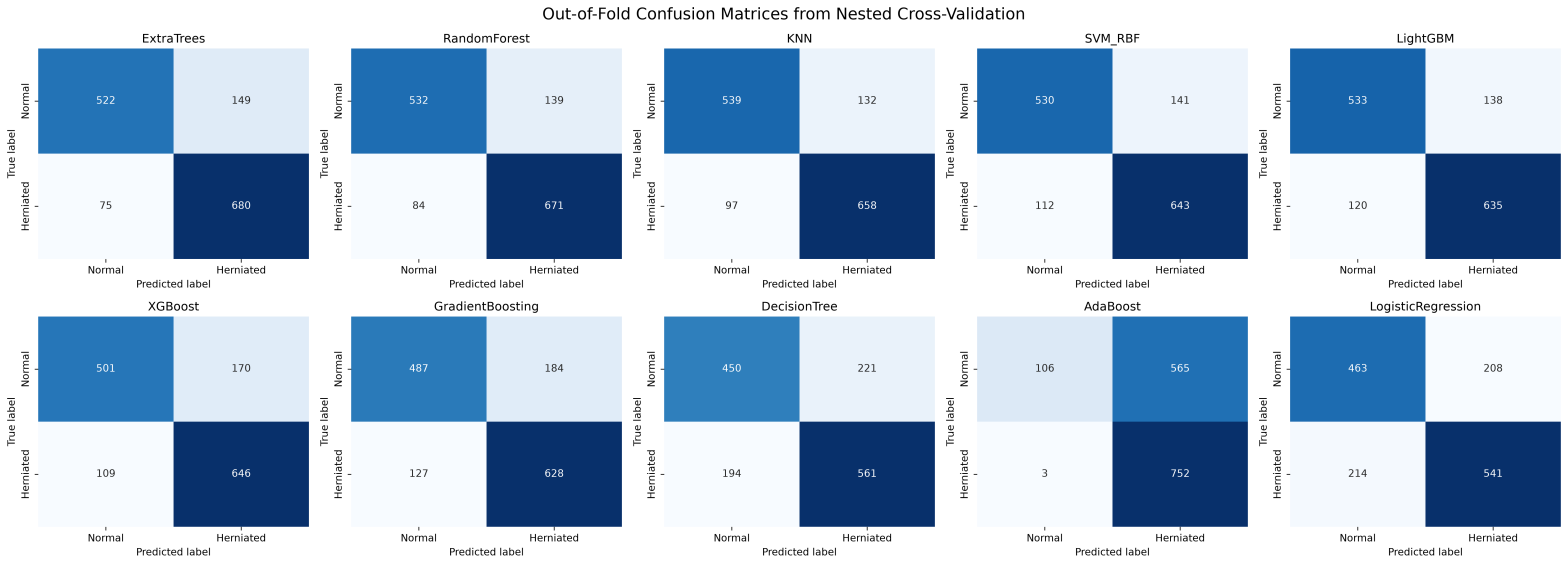
Aggregated out-of-fold confusion matrices for the ten classifiers under five-fold outer nested cross-validation.

Figure 18 presents confusion matrices formed by aggregating the out-of-fold predictions from the five outer folds; consequently, every image contributed exactly one prediction obtained while it was held out from model training. Extra Trees produced 522 true negatives, 149 false positives, 75 false negatives, and 680 true positives. Random Forest produced one additional correct prediction overall and therefore achieved the highest accuracy, whereas Extra Trees produced fewer false negatives and achieved the highest F1-score. KNN yielded the largest number of true negatives among the leading models, consistent with its highest specificity. AdaBoost missed only three herniated images but classified 565 normal images as herniated, illustrating why sensitivity must be interpreted together with specificity. The confusion matrices describe the error distribution but do not independently establish the clinical causes of individual errors; comprehensive case-level review remains necessary for that purpose.

### 4.5. Complementary Performance Visualizations

**Figure 19:**
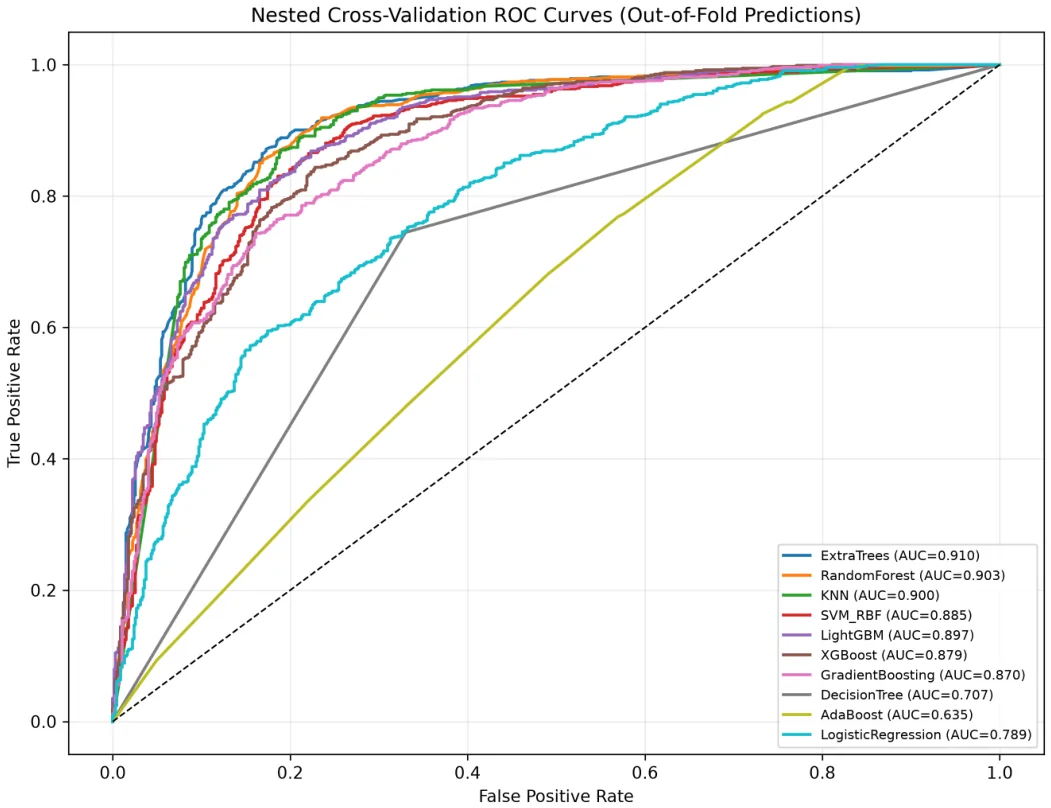
Aggregated out-of-fold ROC curves for the ten classifiers under five-fold outer nested cross-validation.

Figure 19 complements the ROC-AUC values reported in Table 6 by showing the aggregated out-of-fold ROC curves for all ten classifiers under nested cross-validation. Extra Trees achieved the highest ROC-AUC (0.9096), followed by Random Forest (0.9034) and KNN (0.8997), confirming that these models provided the strongest discrimination between normal and herniated MRI images in the evaluated dataset.

**Figure 20:**
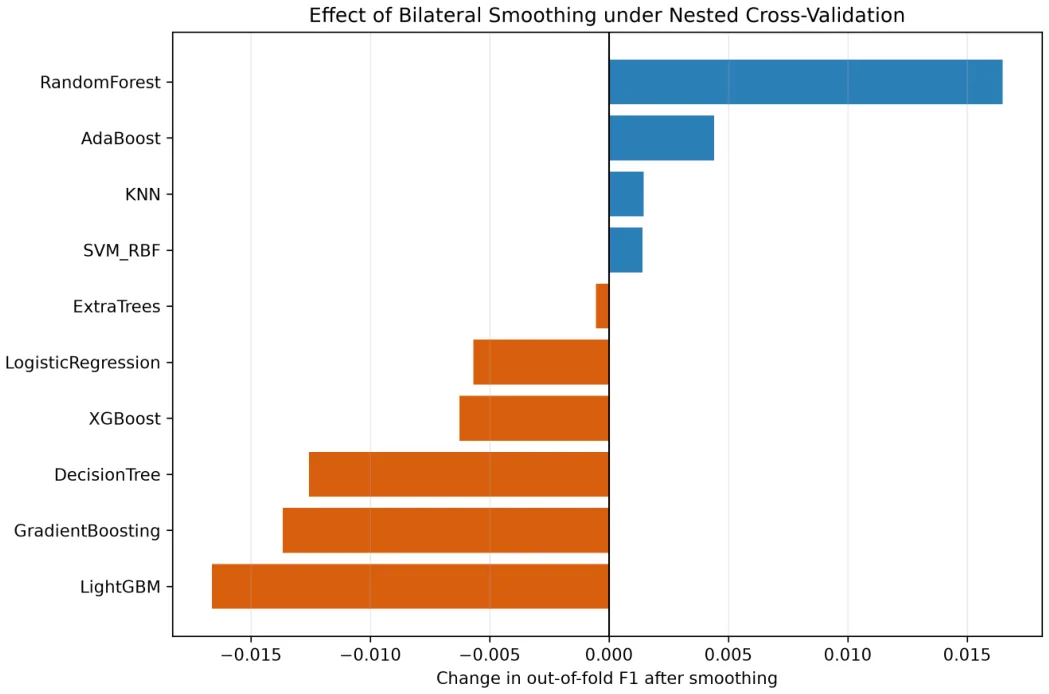
Change in aggregated out-of-fold F1-score after bilateral smoothing for the ten classifiers under nested cross-validation. Positive values indicate improvement after smoothing.

Figure 20 provides a direct visual summary of the paired F1-score differences reported in Table 8. Positive values indicate improvement after bilateral smoothing, whereas negative values indicate a reduction. Random Forest showed the largest improvement (+0.0165), while Extra Trees remained essentially unchanged; the statistical interpretation of these changes follows the bootstrap confidence intervals reported in Table 8.

## 5. Comparative Analysis with Related Work

### 5.1. Overview of Compared Methods

The following will provide a comparison on the methodology to analyse lumbar disc using machine learning-based techniques to the currently existing literature. The key differences between the selection criteria for the examples vary from the type of data being used, whether or not handcrafted/domain-specific features will be used vs. clinical/radiomic information, and the manner that the tasks (classification, localization, outcome prediction, etc.) are measured or represented. The differences in the methodologies highlight the absence of a standardized methodology when comparing classical machine learning approaches in this area of research.

### 5.2. Comparative Table of ML Approaches

The purpose of Table 9 is to assist in structuring this review by summarizing the essential characteristics of the machine learning studies reviewed in this paper. The performance values in Table 9 are presented only as a descriptive overview of the literature and should not be interpreted as direct comparative benchmarks, since the studies used different datasets, feature representations, tasks, and evaluation protocols. (i.e., data modality (MRI, Clin = clinical examination), feature representation, task type, performance).

### 5.3. Comparative Analysis and Key Observations

Table 9 illustrates the notable differences between current machine learning methods for the evaluation of lumbar disc health, including their approaches to features and their objectives. Some studies rely heavily on handcrafted or domain-specific features to assist them in extracting information from MRI scans [26,27,34], which can be texture descriptors or intensity-based measures, as well as morphological features or features extracted—either manually or through other techniques [31]—and the methods for creating those features require complicated pre-processing and skilled design work. However, the proposed framework uses pixel-based features together with pre-processing and Principal Component Analysis (PCA), avoiding the need for manual feature design. In addition, although the accuracy rates of other studies could be higher [26,27], most of their results were derived based on small datasets; therefore, they have limited generalizability. Additionally, previous studies have focused on similar but conceptually distinct problems, disc location [29,32], or prediction of clinical outcome [30]. Therefore, the reported values should be interpreted descriptively rather than as direct benchmarks; the present framework specifically addresses binary image-level classification of normal and herniated lumbar MRI images.

## 6. Discussion

The proposed pipeline, consisting of pre-processing, PCA, and classical ML methods, demonstrated consistent classification performance for lumbar disc MRI images under nested cross-validation. The ablation analysis showed that the effect of smoothing was model-dependent: Random Forest and AdaBoost exhibited supported improvements in F1-score, whereas the changes for the remaining classifiers were small, absent, or uncertain. PCA identified substantial redundancy among the pixel-based features, and the number of retained components was selected independently within each outer fold using only the corresponding inner-validation data. Although 80 components produced the highest aggregated inner-validation F1-score for Extra Trees, this value was not imposed as a single global choice across all final models. Although PCA provides an effective low-dimensional representation, the resulting principal components are mathematically derived features and do not inherently provide direct clinical interpretability. Ensemble classifiers generally produced the strongest results: Random Forest achieved the highest mean accuracy (0.8436), while Extra Trees achieved the highest F1-score (0.8586) and ROC-AUC (0.9096), indicating the strongest overall discrimination among the evaluated models. These findings suggest that tree-based ensembles can capture useful non-linear relationships among the PCA-derived features; however, the performance metrics alone do not establish a specific mechanistic explanation for their relative performance. The confusion matrices characterize the frequency and direction of classification errors but cannot determine whether the misclassified images represent clinically borderline cases; establishing such causes would require case-level radiological review. Another limitation of the present framework is its restriction to binary classification of normal versus herniated MRI images. Although this formulation provides a simplified evaluation setting, it does not distinguish between clinically relevant subtypes or severity levels of lumbar disc pathology. Future work should therefore investigate multi-class classification and grading approaches to better reflect the spectrum of lumbar disc abnormalities encountered in clinical practice. The current framework uses the full sagittal MRI image without explicit disc localization. Although ROI-based analysis may reduce irrelevant information, automated localization must be highly reliable because cropping errors can propagate to the subsequent classification stage, while manual cropping is labour-intensive and difficult to scale. Future work will investigate robust automated disc localization, including the analysis of multiple discs within the same MRI image. While the results of this study demonstrate promising classification performance within the evaluated dataset, the absence of external validation limits conclusions regarding robustness and generalizability to independent clinical populations. Additional work is required to improve feature representation and validate the framework using larger, more diverse datasets, and complementary validation designs. Because patient identifiers were unavailable, patient-level independence across the validation folds could not be verified. Therefore, the reported performance should be interpreted as exploratory rather than confirmatory and requires patient-level and external validation before clinical generalization. Scanner and acquisition metadata were unavailable, precluding scanner-stratified assessment across MRI systems and protocols. This does not alter the reported internal image-level evaluation, but it further limits clinical generalization; external testing with complete patient- and scanner-level metadata is required before conclusions about clinical deployment.

## 7. Conclusions

This research study has developed a comprehensive structured framework for the classification of lumbar discs using MRI, which incorporates methods for pre-processing, dimensionality reduction with PCA, and classical machine learning models to classify. The results show that the effect of smoothing was model-dependent, with supported improvements in F1-score observed for Random Forest and AdaBoost, while PCA reduced redundancy in the high-dimensional pixel-based representation. The number of retained principal components was selected independently within each outer fold using inner validation; 80 components produced the highest aggregated inner-validation F1-score for Extra Trees but were not used as a fixed global choice for all final models. Ensemble classifiers produced the strongest results overall: Random Forest achieved the highest mean accuracy (0.8436), whereas Extra Trees achieved the highest F1-score (0.8586) and ROC-AUC (0.9096). However, external validation on independent datasets is required before broader robustness, generalizability, or clinical applicability can be established. This research demonstrate the potential of classical machine learning with preprocessing and PCA as a computationally efficient framework for binary lumbar MRI classification within the evaluated dataset. There are limitations to this research, including the limited size and variability of the dataset, reliance on pixel-based features, and image-level rather than patient-level nested cross-validation because patient identifiers were unavailable; consequently, overlap between images from the same patient across validation folds cannot be excluded. Future research will focus on the use of more advanced feature representations, improved dataset variability, increased external validation of the classification framework, and improved robustness of classification models for use in real-world clinical settings.

## 8. Future Work

Future studies are able to enhance the performance of this framework by adding advanced feature representations of MRI that show greater anatomical and/or structural detail (e.g., region- or structure-based features) for the purpose of classifying borderline cases. The use of hybrid approaches that include classical machine learning approaches combined with deep feature extractors (including pretrained CNNs) may provide additional classification performance while maintaining computational efficiency. Increasing the sample size and diversity of the dataset through the addition of samples from multiple sites should be done to improve generalization and robustness; external validation is also important for enhancing generalizability. Improvements in the methodologies employed, including automated hyperparameter optimization, ensemble techniques, and patient-level grouped validation when patient identifiers are available, repeated nested cross-validation, and external validation across multiple clinical centres, may provide a more reliable evaluation of the model and will also improve performance. Lastly, continuing to improve interpretability of the model will be vital if it is to be adopted in clinical practice, and identifying the regions and/or features that were most influential in making classification decisions would contribute to an increase in the transparency regarding the use of this model in clinical workflows.

## Figures and Tables

**Table 1:** Dataset distribution used in this study.

Class	Number of Images
Normal	671
Herniated	755
Total	1426

**Table 2:** Mean inner-validation performance of the Extra Trees model across selected PCA component sizes.

Number of Components	Accuracy	F1-Score
10	0.7994	0.8208
20	0.8115	0.8342
40	0.8145	0.8357
60	0.8122	0.8343
80	0.8128	0.8364
100	0.8093	0.8348

**Table 3:** Summary of the evaluated classification models grouped by learning paradigm.

ID	Model Architecture	Algorithmic Category
1	LightGBM	Gradient Boosting Framework (Tree-based)
2	Random Forest	Bagging Ensemble
3	Extra Trees	Extremely Randomized Trees
4	Gradient Boosting	Boosting Ensemble
5	XGBoost	Optimized Gradient Boosting
6	K-Nearest Neighbors (kNN)	Instance-based Learning
7	Support Vector Machine (SVM–RBF)	Non-linear Kernel-based
8	Logistic Regression	Generalized Linear Model
9	Decision Tree	Tree-based Model
10	AdaBoost	Adaptive Boosting

**Table 4:** Hyperparameter settings used for the evaluated classification models.

Model	Hyperparameter Settings
KNN	n_neighbors=3; weights=distance
Extra Trees	n_estimators=400; class_weight=balanced; random_state=42; n_jobs=-1
Random Forest	n_estimators=300; class_weight=balanced; random_state=42; n_jobs=-1
SVM (RBF)	C=2; gamma=scale; kernel=rbf; class_weight=balanced
Logistic Regression	C=1.0; class_weight=balanced; max_iter=2000; random_state=42
Decision Tree	class_weight=balanced; random_state=42
Gradient Boosting	n_estimators=300; learning_rate=0.1; max_depth=3; random_state=42
AdaBoost	n_estimators=200; learning_rate=0.1; random_state=42
XGBoost	n_estimators=400; max_depth=3; learning_rate=0.1; subsample=0.8; colsample_bytree=0.8; eval_metric=logloss; random_state=42; n_jobs=-1
LightGBM	n_estimators=600; num_leaves=31; learning_rate=0.1; random_state=42; n_jobs=-1

**Table 5:** Dataset composition and nested cross-validation design used in the final analysis.

Item	Value
Total images	1426
Normal images	671 (47.05%)
Herniated images	755 (52.95%)
Evaluation unit	Image level
Outer cross-validation	5 folds
Inner cross-validation	3 folds
Candidate PCA components	10, 20, 40, 60, 80, and 100

**Table 6:** Nested cross-validation performance of the ten classifiers.

Model	Accuracy	Precision	Sensitivity	Specificity	F1-Score	ROC-AUC
Extra Trees	0.8429	0.8203	0.9007	0.7779	0.8586	0.9096
Random Forest	0.8436	0.8284	0.8887	0.7928	0.8575	0.9034
KNN	0.8394	0.8329	0.8715	0.8033	0.8518	0.8997
SVM (RBF)	0.8226	0.8202	0.8517	0.7899	0.8356	0.8850
LightGBM	0.8191	0.8215	0.8411	0.7943	0.8312	0.8970
XGBoost	0.8043	0.7917	0.8556	0.7466	0.8224	0.8793
Gradient Boosting	0.7819	0.7734	0.8318	0.7258	0.8015	0.8704
Decision Tree	0.7090	0.7174	0.7430	0.6706	0.7300	0.7073
AdaBoost	0.6017	0.5710	0.9960	0.1580	0.7259	0.6349
Logistic Regression	0.7041	0.7223	0.7166	0.6900	0.7194	0.7891

**Table 7:** Class-wise performance of the best overall model (Extra Trees) based on aggregated out-of-fold predictions.

Class	Precision	Recall	F1-Score
Normal	0.8744	0.7779	0.8233
Herniated	0.8203	0.9007	0.8586
Macro average	0.8473	0.8393	0.8410

**Table 8:** Impact of bilateral smoothing on out-of-fold F1-score under nested cross-validation. Differences are reported with 95% bootstrap confidence intervals.

Model	F1 Before	F1 After	Difference (95% CI)
Extra Trees	0.8591	0.8586	−0.0006 [−0.0114, 0.0104]
Random Forest	0.8410	0.8575	+0.0165 [0.0037, 0.0294]
KNN	0.8503	0.8518	+0.0015 [−0.0085, 0.0120]
SVM (RBF)	0.8342	0.8356	+0.0014 [−0.0047, 0.0075]
LightGBM	0.8478	0.8312	−0.0166 [−0.0334, 0.0005]
XGBoost	0.8287	0.8224	−0.0063 [−0.0260, 0.0133]
Gradient Boosting	0.8152	0.8015	−0.0137 [−0.0330, 0.0058]
Decision Tree	0.7426	0.7300	−0.0126 [−0.0425, 0.0166]
AdaBoost	0.7215	0.7259	+0.0044 [0.0015, 0.0079]
Logistic Regression	0.7251	0.7194	−0.0057 [−0.0165, 0.0049]

**Table 9:** Comparison of classical machine learning approaches.

Study	Data	Method	Total Samples	Herniated Cases	Normal Cases	Features	Performance
Abdullah et al.	MRI	Extra Trees + PCA	1426	755	671	Pixel-based	Acc 0.8429; F1 0.8586
Jafari et al. [25]	Clin	SVM, kNN, LR	400	137	263	Questionnaire	Acc ≈ 0.95
Ghosh et al. [26]	MRI	SVM, kNN, NB	35	Not reported	Not reported	LBP, GLCM	Acc 98.29
Ebrahimzadeh et al. [27]	MRI	SVM, kNN, MLP	210 (discs)	46	164	Intensity, Shape	Acc 97.80
Oktay et al. [29]	MRI	SVM	240 (discs)	Not reported	Not reported	PHOG	Acc ≈ 95
Chiu et al. [30]	MRI + Clin	RF, XGB, SVM	181	Not reported	Not reported	Radiomics	Acc ≈ 0.76
Kocaman et al. [31]	MRI	XGBoost	344	264	80	Morphological	Acc ≈ 0.922
Ghosh et al. [32]	MRI	SVM	318 (discs)	Not reported	Not reported	HOG	Acc ≈ 99
Lin et al. [33]	X-ray	RF, XGB	458	259	199	Disc height	F1 ≈ 0.778
Athertya et al. [34]	MRI	SVM, RF	100	Not reported	Not reported	LBP	Acc ≈ 91.7

## Data Availability

The dataset generated and analyzed during this study is publicly available on Figshare: https://doi.org/10.6084/m9.figshare.32113642. The code used for data analysis is publicly available on GitHub: https://github.com/AbdullahAl-Enezi/Lumbar-Disc-Herniation-Detection (accessed on 23 September 2026). A detailed description of the dataset is available at: https://github.com/AbdullahAl-Enezi/Lumbar-Disc-Herniation-Detection/blob/main/Dataset_Description.pdf (accessed on 23 September 2026).

## Supplementary Materials

The following supporting information can be downloaded at: https://www.mdpi.com/article/10.3390/diagnostics16203288/s1, Table S1: PCA component values selected in the five outer folds. Entries show component value (number of folds). Table S2: Numbers of principal components required to explain 90% and 95% of the variance in each outer-training partition.
