# Supplementary material for: Advancing Lumbar Disc Herniation Classification from MRI Using an Efficient and Reproducible Classical Machine Learning Framework
Source: Diagnostics (Basel). 2026 Oct 10;16(20):3288. doi: 10.3390/diagnostics16203288 (PMC13703152; doi:10.3390/diagnostics16203288)
Supplement: Supplementary file 1 [file diagnostics-16-03288-s001.zip › diagnostics-4508964-supplementary.pdf]

diagnostics

MDPI

Article

Advancing Lumbar Disc Herniation Classification from MRI Using an Efficient and Reproducible Classical Machine Learning Framework

Abdullah Alanazi [1], Meshari Alazmi [1,*], Abdullah Alshammari [1] and Amirah F. Alshammeri [2]

[1] College of Computer Science and Engineering, University of Ha'il, Ha'il 81411, Saudi Arabia;
[2] Diagnostic Radiology Department, College of Medicine, University of Ha'il, Ha'il 81422, Saudi Arabia
* Correspondence: ms.alazmi@uoh.edu.sa

Supplementary Materials

The final evaluation used five outer folds and three inner folds. Standardization and PCA were fitted only on the corresponding training partition. Candidate PCA dimensions were 10, 20, 40, 60, 80, and 100. Tables S1 and S2 report the fold-specific PCA component selections and explained-variance thresholds, respectively. Because consistent patient identifiers were unavailable across the combined multi-source dataset, these results represent image-level rather than verified patient-level validation.

Table S1. PCA component values selected in the five outer folds. Entries show component value (number of folds).

Model	Selected PCA components
Extra Trees	20 (1), 40 (2), 80 (1), 100 (1)
Random Forest	40 (4), 100 (1)
KNN	20 (2), 40 (2), 100 (1)
SVM–RBF	80 (2), 100 (3)
LightGBM	20 (1), 60 (3), 100 (1)
XGBoost	20 (1), 60 (1), 80 (1), 100 (2)
Gradient Boosting	60 (1), 80 (1), 100 (3)
Decision Tree	10 (3), 20 (1), 80 (1)
AdaBoost	60 (2), 80 (3)
Logistic Regression	100 (5)

Table S2. Numbers of principal components required to explain 90% and 95% of the variance in each outer-training partition.

Outer fold	Components for 90%	Components for 95%
1	383	595
2	379	591
3	383	596
4	376	588
5	378	591

Check for updates

Academic Editors: Ayman El-Baz, Asem M. Ali, Ali H. Mahmoud and Moumen Elmelegy

Received: 2 August 2026
Revised: 6 October 2026
Accepted: 7 October 2026
Published: 10 October 2026

Copyright: © 2026 by the authors. Licensee MDPI, Basel, Switzerland. This article is an open access article distributed under the terms and conditions of the Creative Commons Attribution (CC BY) license.

 https://doi.org/10.3390/diagnostics16203288
